# Etiological study of superficial radial nerve neuropathy: series of 34 patients

**DOI:** 10.3389/fneur.2023.1175612

**Published:** 2023-04-20

**Authors:** Lisa B. E. Shields, Vasudeva G. Iyer, Yi Ping Zhang, Christopher B. Shields

**Affiliations:** ^1^Norton Neuroscience Institute, Norton Healthcare, Louisville, KY, United States; ^2^Neurodiagnostic Center of Louisville, Louisville, KY, United States; ^3^Department of Neurological Surgery, University of Louisville School of Medicine, Louisville, KY, United States

**Keywords:** neurology, neurosurgery, superficial radial nerve, Wartenberg’s syndrome, electrodiagnostic studies, nerve conduction studies, electromyography, ultrasound

## Abstract

**Objectives:**

Superficial radial nerve (SRN) neuropathy is a rare focal neuropathy leading to pain and paresthesia of the dorsolateral aspect of the hand. Reported causes include trauma, extrinsic compression, or it may be idiopathic. We describe the clinical and electrodiagnostic (EDX) features of 34 patients with SRN neuropathy of varied etiology.

**Methods:**

This is a retrospective study of patients with upper limb neuropathy referred for EDX studies who were found to have SRN neuropathy based on clinical and EDX findings. Twelve patients also had ultrasound (US) evaluations.

**Results:**

Decreased pinprick sensation was noted in the distribution of the SRN in 31 (91%) patients, and a positive Tinel’s sign was observed in 9 (26%). Sensory nerve action potentials (SNAPs) were not recordable in 11 (32%) patients. Of the patients who had a recordable SNAP, the latency was delayed, and the amplitude was decreased in all cases. Of the 12 patients who underwent US studies, 6 (50%) had an increased cross-sectional area of the SRN at or immediately proximal to the site of injury/compression. A cyst was located adjacent to the SRN in 2 patients. The most common cause of SRN neuropathy was trauma in 19 (56%) patients, of which 15 were iatrogenic. A compressive etiology was identified in 6 patients (18%). No specific etiology was detected in 10 patients (29%).

**Conclusion:**

This study is aimed at raising the awareness among surgeons about the clinical features and varied causes of SRN neuropathy; such knowledge may potentially lessen iatrogenic causes of injury.

## 1. Introduction

Focal neuropathy of the superficial radial nerve (SRN) causes pain, burning, numbness, tingling, and dysesthesias of the dorsolateral aspect of the distal forearm and hand involving the dorsolateral three and a half fingers extending to the distal interphalangeal joint (1–6). Initially reported in a patient with wrist watch compression in 1926 by Schlesigner and Matzdorff, this condition was later referred to as Wartenberg’s syndrome after Wartenberg described 5 patients in 1932 with neuropathy involving the SRN (1–4, 7, 8). Due to the clinical similarities between this condition and meralgia paresthetica (lateral femoral cutaneous nerve neuropathy of the lower extremity) initially reported by Hager in 1885 (9), Wartenberg described SRN neuropathy as cheiralgia paresthetica (1, 10, 11).

The SRN is one of the 2 terminal branches of the radial nerve, derived from the C6 and C7 nerve roots (5). At the elbow, the radial nerve bifurcates into the deep motor branch (posterior interosseous nerve) and superficial, sensory branch (SRN) (2, 11–13). The SRN courses along the radial aspect of the forearm, running beneath the brachioradialis muscle on the lateral side of the radial artery, and piercing the deep fascia at about 9 cm proximal to the radial styloid (2, 5, 10, 11, 13–16). The SRN enters the hand traversing the anatomical snuffbox on the dorsolateral wrist, innervating the lateral two-thirds of the dorsum of the hand and lateral three and a half fingers to the level of the distal interphalangeal joints. The only autonomous sensory region of the SRN is the dorsal web space closest to the thumb (2). Due to the superficial location of the SRN, it is vulnerable to a host of injuries, including trauma (direct blunt trauma to the radial aspect of the forearm which may cause post-traumatic fibrosis/adhesions with neuroma formation, work-related activities with repetitive supination and pronation movements), extrinsic compression (tight wrist watch, handcuffs, plaster cast), intrinsic compression (ganglion cyst, lipoma, abscess, bony spur), iatrogenic (fracture fixation and arthroscopic procedures in the lateral forearm, acupuncture/venipuncture-related injuries, local corticosteroid injections, IV lines/shunts and transradial cardiac catheterization (1–3, 5, 10–12, 15, 17). SRN neuropathy may also be idiopathic (10). Associated conditions comprise de Quervain’s tenosynovitis, carpal tunnel syndrome (CTS), diabetes mellitus, arthritis, ganglions of the first extensor compartment, and flexor carpi radialis tendinitis (2, 3, 6, 10).

Clinical findings consist of sensory abnormalities in the form of decreased pain and touch sensation or allodynia in the dorsolateral hand and dorsal aspect of the thumb and index finger, and a positive Tinel’s sign over the course of the SRN (1, 3–5, 11, 12). There is no motor involvement. Pinching and gripping movements, hyperpronation, and ulnar deviation of the wrist may exacerbate the symptoms. The Finkelstein test (pain on the radial aspect of the wrist when the thumb is flexed into the palm and the wrist is ulnar deviated) may be positive in some cases leading to a mistaken diagnosis of de Quervain tenosynovitis, although it is common for both conditions to coexist (3, 18, 19). Patients with SRN neuropathy often attain symptom resolution with removal of the inciting factor such as a wrist watch, handcuffs, or plaster cast (1–3, 5, 12). Non-surgical management includes rest, splinting, a TENS unit, non-steroidal anti-inflammatory medications, gabapentin or duloxetine, topical application of a lidocaine/gabapentin compound, and minimizing repetitive actions that aggravate symptoms (3). Other treatments prior to surgical intervention include regional nerve blocks proximal to the site of injury or radiofrequency lesioning to block the transmission of potentials towards the spinal cord. A more recent treatment modality is perineural hydrodissection (20). If medical treatment fails, surgery may involve releasing the fascia over the course of the SRN, decompression of the SRN from the radial styloid process, neurolysis of the SRN, or first extensor compartment release if it is associated with de Quervain’s tenosynovitis (2, 3, 5).

One of the most common reasons of referral for EDX studies is pain and paresthesia of the hands. While EDX studies in most patients may show carpal/cubital tunnel syndrome or cervical radiculopathy, occasional cases of focal mononeuropathies of cutaneous nerves of the forearm and hand may be identified. In this report we describe 34 patients with SRN neuropathy diagnosed based on clinical and EDX findings. Ultrasound (US) study of nerves has been available more recently in our facility, and 12 of these patients also underwent US studies. The aim of the current study was to find the etiology of SRN neuropathy in a large cohort of patients referred to our EDX lab from multiple referral sources. The presenting symptoms, clinical and EDX findings, and US features are presented. The varied etiologies and differential diagnosis of SRN are discussed. The significance of EDX and US studies in the diagnostic evaluation of SRN neuropathy is also highlighted.

## 2. Methods

### 2.1. Study population and electrodiagnostic/ultrasound studies

**Figure 1:**
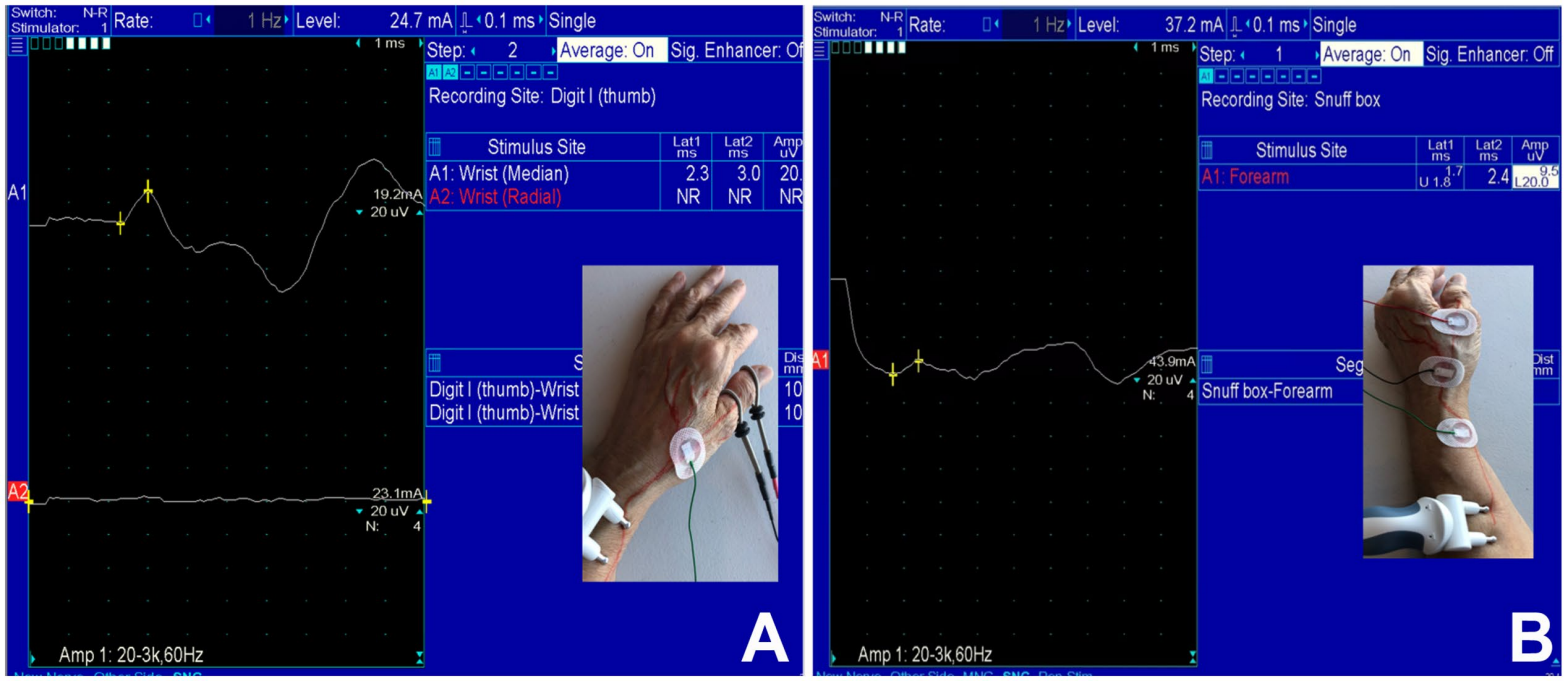
Case 33: **(A)** Absence of sensory nerve action potentials (SNAPs) of the superficial radial nerve (lower trace) and intact SNAP of the median nerve over the thumb (upper trace). **(B)** Recordable SNAP of the SRN with prolonged latency and decreased amplitude over the anatomical snuffbox.

Under an Institutional Review Board (IRB)-approved protocol, we performed a 14-year (July 21, 2008 – November 20, 2022) retrospective analysis of patients referred to our Neurodiagnostic Center for EDX studies to evaluate upper limb neuropathies to identify those with SRN neuropathy. The patients underwent a detailed neurological examination followed by nerve conduction and EMG studies. For the clinical evaluation, patients underwent neurological evaluation including muscle strength, tone, and deep tendon reflexes. They also underwent a sensory examination (pain, temperature, light touch, position sense, and 2-point discrimination at the fingertips). The EDX studies were performed in our American Association of Neuromuscular & Electrodiagnostic Medicine (AANEM)-accredited facility using the standard protocol of our laboratory (21). The SRN was studied by antidromic stimulation with the recording electrode over the anatomical snuff box and/or the thumb (Figures 1A,B). The stimulating electrode was placed 10 cm proximal to the recording electrode. Our EDX lab follows the standard recommendation of keeping the limb temperature at or above 32°C for all nerve conduction studies.

An US study was also conducted using the GE LOGIQ E system and 8–18 MHz probe tracing the SRN from its origin to the anatomical snuff box (similar to the technique described by Chang and colleagues) (22). The cross-sectional area (CSA) of the nerve was measured at different sites along the course of the nerve with a normal range between 1 and 3 mm^2^ in healthy limbs (similar to that reported by Visser and colleagues) (23).

### 2.2. Inclusion criteria

The inclusion criteria for the clinical diagnosis of SRN neuropathy included the following: burning pain, paresthesia, or numbness in the distribution of the SRN; objective sensory loss (hypoesthesia/anesthesia) or allodynia in the distribution of the SRN; and with or without a positive Tinel’s sign in the anatomical course of the SRN. The inclusion criteria for confirmation of SRN neuropathy by EDX studies included: absent sensory nerve action potentials (SNAP) or delayed peak latency, and/or reduced SNAP amplitude. Several metrics were collected including age and gender of the patient, symptom laterality (right/left), clinical history and neurological examination findings, EDX findings, and US features.

### 2.3. Exclusion criteria

The exclusion criteria included patients with clinical/EDX features of polyneuropathy from varied etiologies. Patients with additional features suggestive of more proximal radial nerve or brachial plexus involvement were also excluded.

### 2.4. Ethical approval and informed consent

Informed consent was obtained from all patients. The IRB determined that our study was exempt according to 45 CFR 46.101(b) under Category 4. The IRB number is 22.1024.

## 3. Results

A total of 34 patients were diagnosed with SRN neuropathy based on presenting symptoms, findings on neurological examination, and EDX studies (Table 1). The mean age was 50 years (range: 31–69 years), and the majority (21 [62%]) was female. The SRN neuropathy occurred on the right side in 21 (62%) patients, the left side in 12 (35%), and bilateral in 1 (3%) case. Four patients had concurrent diabetes mellitus.

**Table 1:** Demographics, presenting symptoms, neurological examination, and electrodiagnostic studies of patients with superficial radial nerve neuropathy referred for electrodiagnostic studies at our facility.

Patient #	Age (years)/Gender	Side (R/L/B)	Cause	Presenting Symptoms at time of EDX	Neurological Examination at time of EDX	EDX findings Wrist-Thumb latency, amplitude/mid forearm-snuff box latency, amplitude
1	54/F	R	Idiopathic	Numbness thumb; pain wrist	Decreased pinprick sensation R thumb	2.5, 8.3/2.4, 15.9
2	50/F	R	Compression from cyst	Pain, paresthesia thumb; limited mobility thumb	Tender IP joint thumb; decreased pinprick sensation radial 2 digits	2.5, 7.8/ND
3	41/F	L	Iatrogenic: surgery for de Quervain’s synovitis	Pain thenar area, index/middle fingers	Decreased pinprick sensation thumb; hyperalgesia over radial styloid and snuff box area. Positive Tinel	2.9, 7.5/ND
4	36/M	R	Idiopathic	Numbness of thumb, index finger	Decreased pinprick sensation thumb and index finger	2.7, 3.4/2.1, 10.2
5	64/M	R	Trauma: moved heavy object	Numbness and shock-like sensation dorsal aspect of thumb, index finger	Decreased pinprick sensation anatomical snuffbox; positive Tinel’s sign over distal radius	NR/NR
6	58/F	R	Iatrogenic: radial tunnel surgery	Pain, numbness, tingling radial forearm to wrist	Tingling sensation over anatomical snuffbox, radial distal forearm; tender radial distal forearm/ brachioradialis muscle	ND/2.4,9.5
7	60/F	L	Trauma: moved heavy object Diabetic	Pain radial distal forearm at snuffbox; paresthesia thumb, index finger	Tender radial wrist and allodynia with positive Tinel’s sign; decreased pinprick sensation thumb and index finger	2.6, 10.4/ND
8	43/F	L	Compression: wore Fitbit and typed for several hours	Pain/burning wrist, dorsal aspect of thumb, index finger	Decreased pinprick sensation thumb and index finger/ anatomical snuffbox	2.6, 12.5/2.2, 28.8
9	62/M	R	Iatrogenic: rotator cuff repair Diabetic	Burning sensation/pain radial wrist, hand	Decreased pinprick sensation anatomical snuff box	NR/2.6, 4.4
10	42/F	R	Idiopathic	Paresthesia radial forearm/thumb, index finger	Positive Tinel’s sign over distal radius	2.7, 3.4/2.4, 21.8
11	31/M	R	Idiopathic	Numbness thumb	Decreased pinprick sensation thumb	NR/NR
12	54/M	R	Idiopathic	Numbness thumb	Decreased pinprick sensation thumb and index finger	NR/NR
13	40/F	L	Idiopathic	Numbness/tingling thumb, wrist, forearm	Decreased pinprick sensation radial 4 digits, anatomical snuffbox, dorsum hand	NR/NR
14	60/F	R	Idiopathic	Paresthesia snuffbox; pain radial distal forearm to elbow	Decreased pinprick sensation over distal radius and anatomical snuffbox	NR/NR
15	49/M	R	Idiopathic	Numbness lateral distal forearm/ snuffbox/thumb, index finger	Decreased pinprick sensation anatomical snuff box, thumb	2.9, 1.2/ 2.3, 12.5
16	65/F	R	Iatrogenic: radial tunnel release	Paresthesia, pain lateral forearm, snuff box	Decreased pinprick sensation over radial side of distal forearm, anatomical snuff box and thumb, index finger	NR/2.5, 7.6
17	67/F	R	Iatrogenic: reverse shoulder replacement	Pain, paresthesia forearm, snuff box, thumb, index finger	Allodynia over anatomical snuff box	NR/NR
18	37/F	R	Trauma: laceration injury of forearm	Pain, paresthesia radial side of wrist, hand	Decreased pinprick sensation over thumb	ND/NR
19	32/M	L	Iatrogenic: Phlebotomy	Pain, paresthesia thumb, index, middle fingers	Decreased pinprick sensation over thumb and index finger; Positive Tinel	NR/ND
20	49/F	R	Compression while in coma	Pain, paresthesia over distal forearm	Decreased pinprick sensation over distal radial forearm and thumb	NR/ND
21	69/F	R	Iatrogenic: CMC joint surgery Diabetic	Pain, paresthesia thumb, index finger	Decreased pinprick sensation over thumb	NR/2.5, 9.8
22	69/F	R	Iatrogenic: repair of fracture of radius	Numbness thumb	Decreased pinprick sensation over thumb	NR/ND
23	46/M	R	Compression after being handcuffed	Pain, paresthesia distal radial forearm	Decreased pinprick sensation over distal radial forearm and thumb; positive Tinel	2.7, 12.4/2.4, 13.6
24	41/M	L	Trauma: airbag injury	Paresthesia of thumb, index and middle fingers	Decreased pinprick sensation over thumb	2.6, 4.5/2.2, 13.6
25	48/F	L	Idiopathic	Pain, paresthesia digits 1 & 2	Decreased pinprick sensation snuff box, thumb, index finger	2.4, 7.2/2.4, 13.3
26	37/F	L	Iatrogenic: Injury from hand restraint while in ICU	Paresthesia wrist, hand	Decreased pinprick sensation thumb	NR/ND
27	52/F	R	Iatrogenic: Phlebotomy; extravasation of IV fluid. Diabetic	Numbness of thumb and distal radial forearm	Decreased pinprick sensation over thumb and radial wrist	NR/NR
28	60/M	L	Iatrogenic: Biceps tendon repair	Numbness of thumb	Decreased pinprick sensation over thumb and anatomical snuff box	NR/NR
29	51/M	R	Iatrogenic: Biceps tendon repair	Paresthesia of radial side of hand	Decreased pinprick sensation over thumb, radial wrist, forearm	NR/NR
30	53/F	L	Iatrogenic: surgical repair of CMC joint of thumb	Pain and paresthesia over anatomical snuffbox	Allodynia over the base of thumb and anatomical snuffbox; loss of pinprick sensation over dorsolateral wrist; positive Tinel	NR/NR
31	36/M	L	Iatrogenic: Excision of ganglion cyst	Pain and paresthesia of radial wrist	Decreased pinprick sensation over the thumb	2.6, 5.2/3.4, 3.9
32	40/F	L	Iatrogenic: Excision of granular cell tumor in the proximal forearm	Pain in forearm numbness of radial forearm and thumb	Decreased pinprick over radial forearm, wrist and thumb and index finger	2.4, 4.3/2.6,13.0
33	64/F	R	Compression by ganglion cyst	Pain wrist and paresthesia of thumb and index finger	Ganglion cyst at volar radial wrist; decreased pinprick over anatomical snuffbox and thumb	NR/2.4, 9.5
34	58/F	L, R	Idiopathic	Painful paresthesia of left hand, numbness of right hand	Decreased pinprick over the dorsolateral forearm and anatomical snuffbox area, L > R; positive Tinel on L	L: NR/NR R: NR/2.2, 11.9

EDX, electrodiagnostic; R, right; L, left; B, bilateral; CTR, carpal tunnel release; IP, Interphalangeal; CMC, carpometacarpal joint; Sup Rad N, superficial radial nerve; PT, physical therapy; CSA, cross-sectional area; ND, not done; NR, not recordable (Absent SNAP); Iatro, iatrogenic; ICU, intensive care unit. Normal Data: cut off values: Wrist to thumb (10 cm): Peak latency 2.2 ms, Peak to peak amplitude: 10 μV. Midforearm to snuff box (10 cm): Peak latency 2.0 ms, Peak to peak amplitude: 20 μV.

### 3.1. Clinical findings of patients with SRN neuropathy

Thirty-one (91%) patients described numbness/tingling/paresthesia in the distribution of the SRN, while 18 (53%) patients experienced pain/burning/hypersensitivity in this location. On exam, decreased pinprick sensation was noted in the distribution of the SRN in 31 (91%) patients, and a positive Tinel’s sign was observed in 9 (26%). No muscle weakness of the upper extremities was detected.

### 3.2. Electrodiagnostic studies in patients with SRN neuropathy

The results of the EDX studies are summarized in Table 1. SNAPs of the radial nerve from both the thumb and from the wrist were not recordable in 11 (32%) patients. Of the patients who had a recordable SNAP, the latency was delayed, and the amplitude was decreased in all cases. Figure 1 (Case 33) demonstrates absent SNAP over the thumb and intact SNAP over the wrist.

### 3.3. Ultrasound studies in patients with SRN neuropathy

**Figure 2:**
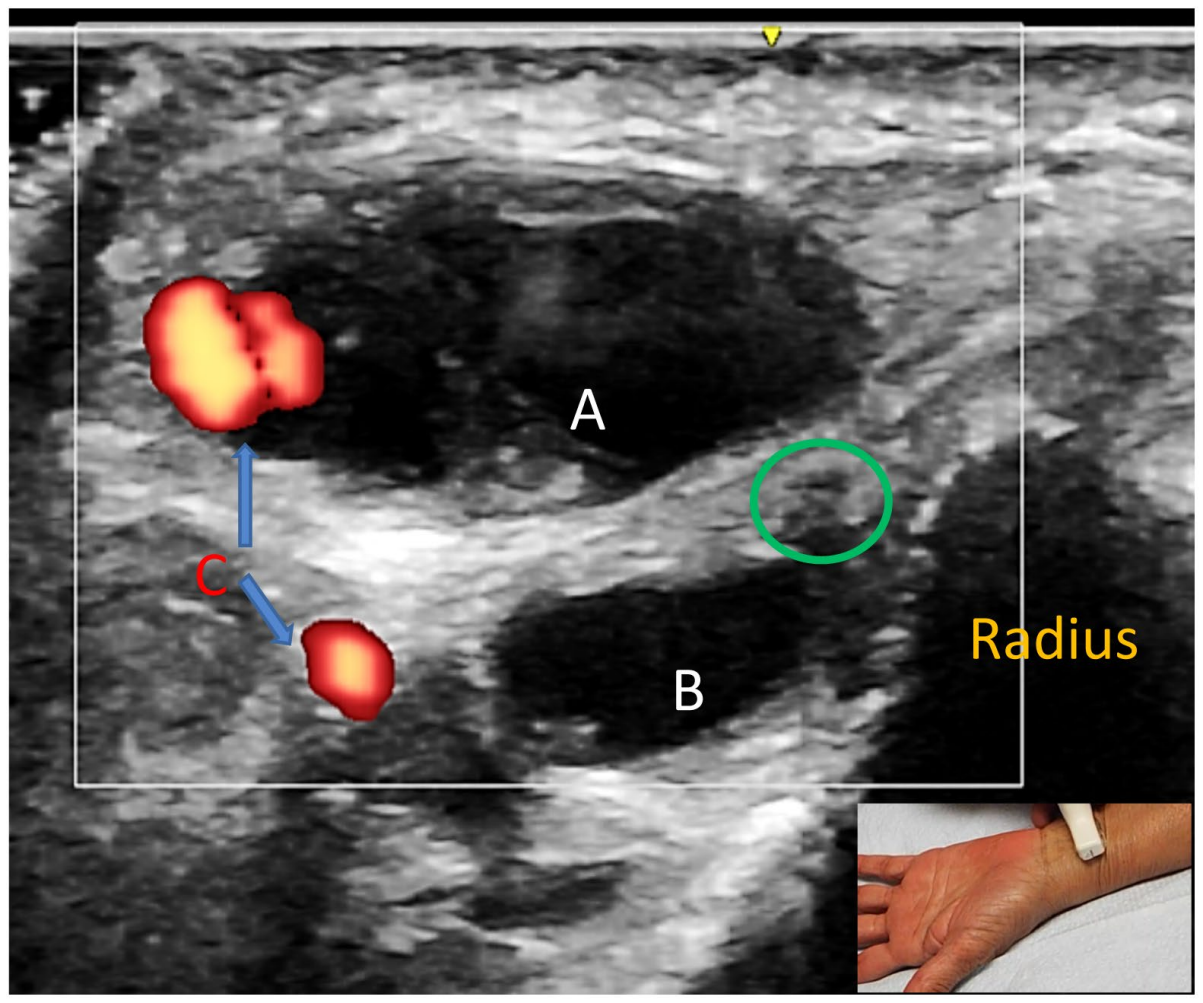
Case 33: Short axis view proximal to the wrist showing bilobed cyst **(A,B)** and the superficial radial nerve (green circle). Color Doppler shows blood vessels (orange) close to the cyst **(C)**.

Of the 12 patients who underwent US studies, 6 (50%) had an increased CSA at or immediately proximal to the site of injury/compression in the distal forearm, while the CSA was normal in 6 (50%) patients. The actual CSA in the abnormal nerves ranged from 3–5 mm^2^, and values in the “non-expanded” part ranged from 2–3 mm^2^. A cyst was located adjacent to the SRN in 2 patients (Cases 2 and 33; Figure 2).

### 3.4. Etiology of SRN neuropathy

In 19 (56%) of the 34 cases, trauma was identified as the cause: iatrogenic in 15 patients and external trauma in 4 (moving heavy objects [2], laceration injury to forearm [1], airbag injury [1]). The iatrogenic injury was caused by radial tunnel release (2), phlebotomy (2), carpometacarpal joint (CMC) joint surgery (2), biceps tendon repair (2), surgery for de Quervain’s synovitis (1), rotator cuff repair (1), reverse shoulder replacement (1), excision of ganglion cyst (1), injury from hand restraint while in ICU (1), excision of a granular cell tumor in the proximal forearm (1), and repair of a radial fracture (1). Extrinsic compression was the cause in 3 (9%; wearing a Fitbit and typing for several hours [1], handcuffs [1], and prolonged pressure on the forearm while comatose [1]). Intrinsic compression from a cyst near the SRN was the cause in 2 (6%). No specific etiology could be detected in 10 (29%) of patients.

## 4. Discussion

A thorough clinical history and comprehensive neurological examination of the upper extremities are important initial steps in the evaluation of a patient with pain and altered sensation in the dorsolateral forearm, hand, and fingers. Several conditions should be considered in the differential diagnosis of dorsoradial wrist pain, including de Quervain’s tenosynovitis, ganglion cysts, joint sprain, lateral antebrachial cutaneous nerve injury, CTS, cervical radiculopathy, brachial plexopathy, and arthritis (1, 3, 5, 6, 11). The diagnosis of SRN neuropathy is often delayed or misdiagnosed as de Quervain’s disease. A positive Finkelstein’s test is more typically seen in de Quervain’s disease (3, 19), but patients with SRN neuropathy may also experience exacerbation of pain (18). Selective nerve block of the SRN with lidocaine may be helpful to confirm SRN neuropathy and differentiate it from lateral antebrachial cutaneous nerve neuropathy (3). Uncovering the etiology of the pain and sensory abnormalities in an expedited manner is crucial, as removal of the compression has the potential to resolve the symptoms.

EDX studies are necessary for confirming SRN neuropathy following the history and physical examination. Nerve conduction tests detect the location of the nerve injury and provide clues to the underlying pathology: demyelination, conduction block due to axonal injury. Slowing of conduction velocity indicates demyelination while loss or decrease in the amplitude of SNAP suggests axonal loss (12). The data should include SNAP latency, amplitude and preferably comparison between the right and left sides. If a distal lesion is suspected, recording from the branches to the thumb should be performed (24).

US offers a valuable adjunct in the diagnosis of SRN neuropathy; it is readily available at the point of care, inexpensive, painless, and without radiation exposure (12, 16). US provides anatomical details that are not available through EDX studies by providing insight into potential pathologies within nerve structures (12, 13, 16). US provides data on CSA, overall echogenicity, echotexture, and fascicle size along the course of the SRN (13). It may reveal the underlying etiology of SRN neuropathy, including ganglion cysts (as in Case 33), masses and tumors, entrapment from surrounding tissues, vascular structures, and neuromas (13). In Umay and colleagues’ study of 138 patients with hand osteoarthritis, they investigated whether these patients had concurrent SRN neuropathy (25). US was utilized to reveal the presence of synovitis in the first CMC joint. Both synovitis in the 1^st^ CMC joint and increased CSA of the first extensor compartment of the wrist were independent risk factors for reduced conduction velocity in SRN neuropathy.

Several comprehensive studies on SRN neuropathy have been reported (3, 7, 8, 26, 27). In Lanzetta and colleagues’ study of 50 patients (52 cases) of SRN neuropathy, most patients were female (80%) with an average age of 45 years (20–69 years) (8). The primary cause was trauma, with watch strap compression in 8 patients. All patients had pain, numbness/dysesthesia of the dorsoradial aspect of the hand. De Quervain’s disease was associated with 50% of the cases; CTS accompanied 8/52 (15%) cases. EDX studies were performed in 12 (23%) cases, of which 6 were normal. Decreased sensory conduction was present in 2 cases, and an underlying polyneuropathy was detected in 2 others. In Dellon and colleagues’ study of 51 patients (58 “entrapments”) with SRN neuropathy, the mean age was 42 years (19–62 years), with a female predominance (69%) (7). A total of 58% of patients were cases related to Workman’s Compensation, with another 13% work-related. Only 4 (8%) patients had no work or trauma basis for their symptoms. CTS and diabetes mellitus were associated with the SRN neuropathy in 57 and 10% of patients, respectively. EDX studies were conducted in 14 (27%) patients (19 cases), 3 of which were normal. A decreased conduction velocity was observed in 8 patients, and there was a decreased amplitude in 5 patients. No sensory recording was obtainable in 3 patients. These authors suggest that patients with an underlying neuropathy such as diabetes mellitus may be predisposed to nerve entrapment in areas with an anatomic predisposition to nerve entrapment without a particular history of trauma or injury (7). In Kong and colleagues’ review of 7 case series and 23 case reports of SRN neuropathy, the average patient age was 41 years, and there was a higher proportion of females (3). The most common causes were work-related complaints, blunt trauma, and direct external compression (watch straps, handcuffs, and casts/splints). Associated conditions included de Quervain’s tenosynovitis, CTS, and diabetes mellitus.

While our study concurs with the existing literature on SRN neuropathy with respect to a higher proportion of females, the mean age (50 years) of our patients was substantially higher and it was idiopathic in one-third of patients. Of the 15 patients with an iatrogenic etiology, 2 had shoulder surgeries, specifically a rotator cuff repair and a reverse shoulder replacement immediately prior to the onset of SRN neuropathy. Literature review showed only 1 previous publication reporting SRN neuropathy after shoulder surgery (28); in that report Singh and colleagues described a 42-year-old female who underwent an arthroscopic rotator cuff repair of the right shoulder. The surgery was performed in the beach chair position with the operated side supported by an arm support; the surgery done under interscalene block, and the operative time was 2 h. Postoperatively, the patient complained of pins, needles, and paresthesia in the distribution of the SRN, and an isolated sensory deficit of this nerve was detected. Initially her symptoms were thought to be due to the nerve block. After 6 months of continued symptoms, nerve conduction studies revealed an isolated lesion of the SRN 18 cm proximal to the radial styloid. Their conclusive diagnosis was positioning induced pressure injury of the SRN. These authors postulate that the SRN neuropathy was due to prolonged compression of the forearm against a poorly padded arm side support (28). We believe that the positional mechanism of injury of the 2 patients who underwent shoulder surgeries in our study was similar to that reported by Singh and colleagues. Patient #9, a diabetic, underwent a right shoulder arthroscopic capsular release with rotator cuff debridement, subacromial decompression and acromioplasty, and chondroplasty. He received a shoulder interscalene block followed by general endotracheal anesthesia. The patient’s right arm was placed in 12 pounds of inline traction throughout the duration of the surgery. The patient complained of numbness of the right forearm, wrist, and thumb postoperatively. The symptoms persisted 3 months and, similar to Singh and colleagues’ case, were attributed by the surgeon to the nerve block. Patient #17 underwent a total reverse shoulder arthroplasty. She was also administered a scalene block followed by general anesthesia. She was subsequently placed in a beach chair position. The patient always wore an abduction sling postoperatively. The patient reported pain of her right thumb as well as pain and numbness of the first dorsal webspace at her first follow-up appointment 17 days after surgery. The surgeon presumed that these symptoms were due to the sling use, and it was discontinued. The symptoms persisted 1 month later despite the sling termination. The scalene block during surgery was then considered as the more likely culprit. The patient continued to experience painful paresthesia in the distribution of the SRN 22 months later. Both patients in our study who underwent shoulder surgery most likely sustained SRN injury from compression neuropathy intraoperatively. These cases illustrate the importance of anatomic knowledge of the SRN and additional safety measures especially in high-risk patients with systemic medical conditions to avoid iatrogenic injury to the SRN during surgeries.

In two of our patients, iatrogenic injury occurred during repair of biceps tendon rupture at the antecubital fossa. While the lateral antebrachial cutaneous and posterior interosseous nerves are more often injured, it is important to locate and protect all the nerves, including the SRN during biceps tendon repair. There were 2 patients who also developed SRN neuropathy after phlebotomy which should be considered an avoidable complication; avoiding sites where the cephalic vein is close to SRN has been stressed by Kim et al. (16).

The strength of the present study is the large number of patients with a SRN neuropathy confirmed by EDX studies with the associated use of US in many cases. An US study contributes to medical care as it is an easily accessible test and can be done serially. The study will increase the awareness of SRN neuropathy especially in situations where iatrogenic injury can occur. A limitation of the study is its retrospective nature. Another limitation is the lack of follow-up after the patients’ EDX evaluations, and we do not have data on the treatment of these patients. This has prevented our ability to evaluate their long-term outcome.

## 5. Conclusion

Surgeons should be aware of the rare condition of SRN neuropathy and the associated diverse etiologies. EDX and US studies play important roles in the evaluation of pain and sensory abnormalities in the distribution of the SRN. Specific precautionary measures including identification and protection of the nerves should be taken in the surgical setting to minimize injury to the SRN.

## Data availability statement

The original contributions presented in the study are included in the article/supplementary material, further inquiries can be directed to the corresponding author.

## Ethics statement

The University of Louisville Institutional Review Board determined that our study was exempt according to 45 CFR 46.101(b) under Category 4. The IRB number is 22.1024. The patients provided their written informed consent to participate in this study.

## Author contributions

LS, VI, and CS: conceptualization and data collection and curation. LS and CS: project administration. LS and VI: formal analysis, interpretation, methodology. LS: writing – original draft. LS, VI, YZ, and CS: writing – review and editing. All authors contributed to the article and approved the submitted version.

## Conflict of interest

The authors declare that the research was conducted in the absence of any commercial or financial relationships that could be construed as a potential conflict of interest.

## Publisher’s note

All claims expressed in this article are solely those of the authors and do not necessarily represent those of their affiliated organizations, or those of the publisher, the editors and the reviewers. Any product that may be evaluated in this article, or claim that may be made by its manufacturer, is not guaranteed or endorsed by the publisher.
